# Intermittent Wolff-Parkinson-White

**DOI:** 10.11604/pamj.2018.30.290.15317

**Published:** 2018-08-24

**Authors:** Mickyas Aberham, Jeffrey John Thompson

**Affiliations:** 1 MCM General Hospital, Addis Ababa, Ethiopia; 2 State University of New York at Buffalo, Department of Emergency Medicine, New York, USA

**Keywords:** Wolff-parkinson-white, ECG, arrhythmia

## Image in medicine

A 16-year-old female presented to the emergency department with complaints of palpitations of two hours duration. She had similar episodes in the past brought on by stress, anger, chocolate, or coffee. The ECG obtained in the course of her medical work-up led to the diagnosis of Wolff-Parkinson-White (WPW) syndrome. The characteristic short PR segment and delta wave are best seen in the lead I rhythm strip. In this rare example, the electrocardiographic morphology alternates between multiple beats of WPW and beats of normal sinus rhythm (A). It is important to recognize the characteristic features of pre-excitation syndromes such as WPW as patients may require treatment with antiarrhythmic drugs and/or radiofrequency ablation. Furthermore, emergency management of tachyarrhythmias in patients with a known history of WPW may differ from those without. However, as demonstrated in this example, such findings may be intermittent thus making an accurate and timely diagnosis challenging. Once it is apparent, proper documentation with copies of the ECG or rhythm strip in the patient record is imperative. In this case, the patient was also provided with a copy of her ECG to show the cardiologist which proved to be important since a repeat ECG at the cardiology clinic later the same day was unremarkable (B).

**Figure 1:**
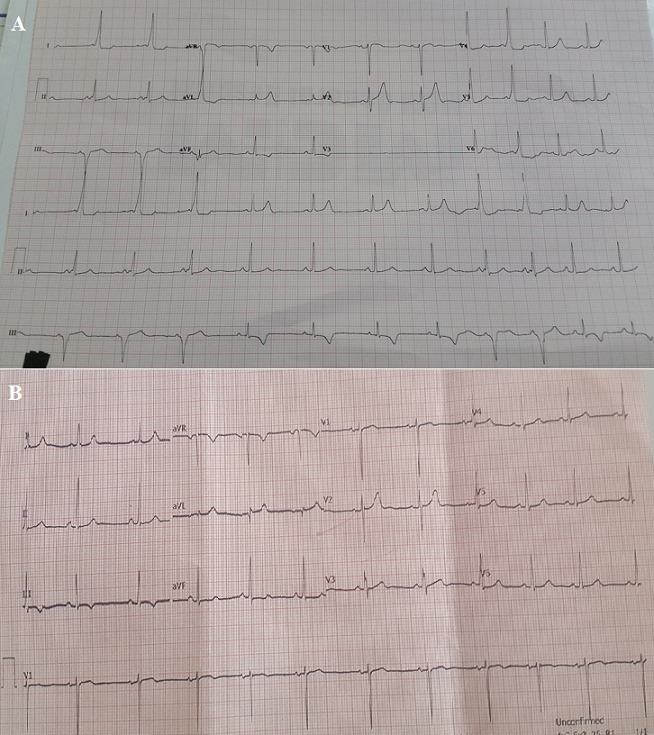
Intermittent WPW and normal ECG

